# The Role of Social Isolation in Shaping Health Outcomes in Older Adults: A Regional Analysis

**DOI:** 10.1093/geroni/igaf122.4217

**Published:** 2025-12-31

**Authors:** Vince Falco, Lynn Wiles, Jonathan Romero, Leorey Saligan

**Affiliations:** Old Dominion University, Virginia Beach, Virginia, United States; Old Dominion University, Virginia Beach, Virginia, United States; Old Dominion University, Virginia Beach, Virginia, United States; Rutgers University, Newark, New Jersey, United States

## Abstract

Social isolation is a critical public health concern in older adults and is linked to a range of adverse health outcomes across diverse sociodemographic groups. This study investigated the associations between social isolation with physical and mental health of older adults residing in Hampton Roads, a racially diverse region in Southeastern Virginia, home to a large population of senior military veterans. Secondarily, the study explored how social determinants of health (SDoH) and comorbid medical conditions influenced the relationship of social isolation with physical and mental health of these seniors. Physical and mental health were measured using the Patient-Reported Outcomes Measurement Information System Global Health short form. Social isolation was assessed using the Social Connection and Isolation portion of the National Institutes of Health Social Determinants of Health form. The SDoH included in this analysis as covariates were individual-level sociodemographic data, such as age, sex, race, marital status, highest level of education completed, current employment status and income, all collected via self-report. Comorbidities were assessed using the Charlson Comorbidity Index. Stepwise regression analysis of 174 participants with complete data showed that each one-unit increase in social isolation score (less socially isolated) was associated with a 1.16-point improvement in mental health scores, after adjusting for all covariates. Social isolation accounted for 2.2% of the variance in mental health outcomes. No significant association was found between social isolation and physical health using the same analytical approach. Interventions to reduce social isolation may be a potential strategy to improve mental well-being of older adults.

